# Parental attitudes and opinions on the use of psychotropic medication in mental disorders of childhood

**DOI:** 10.1186/1744-859X-6-32

**Published:** 2007-11-15

**Authors:** Helen Lazaratou, Dimitris C Anagnostopoulos, Elias V Alevizos, Fotini Haviara, Dimitris N Ploumpidis

**Affiliations:** 1 Department of Psychiatry, Community Mental Health Center Byron-Kesariani, University of Athens, 14 Dilou St.,16121 Athens, Greece; 2 Department of Neurology, Children's General Hospital of Athens "Agia Sofia", Thivon & Papadiamantopoulou St., 11527 Athens, Greece

## Abstract

**Background:**

The limited number of systematic, controlled studies that assess the safety and efficacy of psychotropic medications for children reinforce the hesitation and reluctance of parents to administer such medications. The aim of this study was to investigate the attitudes of parents of children with psychiatric disorders, towards psychotropic medication.

**Methods:**

A 20-item questionnaire was distributed to 140 parents during their first contact with an outpatient child psychiatric service. The questionnaire comprised of questions regarding the opinions, knowledge and attitudes of parents towards children's psychotropic medication. Sociodemographic data concerning parents and children were also recorded. Frequency tables were created and the chi-square test and Fisher's exact tests were used for the comparison of the participants' responses according to sex, educational level, age and gender of the child and use of medication.

**Results:**

Respondents were mostly mothers aged 25–45 years. Children for whom they asked for help with were mostly boys, aged between 6 and 12 years old. A total of 83% of the subjects stated that they knew psychotropic drugs are classified into categories, each having a distinct mechanism of action and effectiveness. A total of 40% believe that there is a proper use of psychotropic medication, while 20% believe that psychiatrists unnecessarily use high doses of psychotropic medication. A total of 80% fear psychotropic agents more than other types of medication. Most parents are afraid to administer psychotropic medication to their child when compared to any other medication, and believe that psychotherapy is the most effective method of dealing with every kind of mental disorders, including childhood schizophrenia (65%). The belief that children who take psychotropic medication from early childhood are more likely to develop drug addiction later is correlated with the parental level of education.

**Conclusion:**

Parents' opinions and beliefs are not in line with scientific facts. This suggests a need to further inform the parents on the safety and efficacy of psychotropic medication in order to improve treatment compliance.

## Background

Although psychotropic medications have been used for the treatment of psychiatric disorders for more than 50 years, less attention has been given to their utilization for the treatment of psychiatric disorders in children and adolescents. The limited number of systematic, controlled studies to assess the safety and efficacy of psychotropic medications for children, and the psychodynamic orientation of the majority of child psychiatrists [1,2], reinforces the hesitation and reluctance of parents to administer such medications to their children.

Clinical trials in children raise methodological problems, such as the forming of homogeneous groups due to the difficulty in defining diagnostic criteria and the measurement of the changes that the treatment has caused [3]. Additionally, they raise clinical, moral and legal dilemmas – and as a consequence controlled, double-blind studies and obliging child psychiatrists to draw information from them concerning the efficacy and safety of medication from open studies remain few and far between in child psychiatry[4]. The result of the aforementioned difficulties is the rarity of data concerning pharmacokinetics and pharmacodynamics of psychotropic agents in children, even though there are different characteristics of metabolism (e.g. faster absorption, shorter duration of therapeutic blood levels).

In some countries, including our own [5], the lack of research on the efficacy of psychotropic medication on mental disorders of childhood contributes to the child psychiatrist's hesitation to suggest drug treatment. The deficit of studies poses a real dilemma for the child psychiatrist; whether to refuse to administer a treatment that might prove beneficial, or to accept responsibility for safety. Child psychiatrists are also affected by the existing negative attitudes of the public, and their hesitation, in turn, affects the acceptance of drug treatment by the parents.

In an earlier French study [1], it was reported that 70% of child psychiatrists were reluctant to prescribe psychotropic medication. More recent data however, show that the use of psychotropic medication in children has significantly increased during the past few years [6]. In the United States, the consumption of antipsychotic drugs nearly doubled between 1996 and 2001 in patients aged 2–18 years, with an increase of 61% for preschool ages, 93% for the ages between 6 and 12, and 116% between 13 and 18 years of age [7]. More recent studies [8,9] confirm that the frequency of psychotropic prescribing in child psychiatry continues to increase. Antipsychotics, in particular the atypical ones, have been used at an increasing rate over the past few years, and they have frequently been used to treat externalized, non-psychotic disorders [8,10]. Polypharmacy is also on the rise. Multiple psychotropic medication use occurred in nearly one third of youths with any psychotropic treatment [11,12].

While the provision of medication is often determined by ideological, political and social factors, it has been claimed that the mistaken perceptions on the acting mechanisms of psychotropic medication is not related to the socio-economic status or education level of the families. An important factor that may change the attitude of the parents is whether they themselves would accept medication [13,14].

In the literature there are only a few reports regarding the attitudes and opinions of the general public and the medical community about psychotropic medication. An earlier Greek study of a general population sample and non-psychiatric physicians illustrates a negative view of psychotropic drugs and psychiatric treatment regarding safety and efficacy, which affects the scientific evaluation of psychotropic medication and may have negative consequences on their therapeutic application. The opinion that psychotropic medications cause dependency and physical damage, when administered over a long period of time, and that they cause alterations of personality, are just some among the views expressed [15].

The aim of the current study was therefore to investigate the opinions on and attitudes toward psychotropic medication of parents of children with psychiatric disorders who are users of a child psychiatry service.

## Methods

### Materials

Study subjects were 134 out of 140 (90.3%) parents of an equal number of children and adolescents under 18 years of age. All were residents of the Byron-Kesariani area of the city of Athens, Greece.

### Methodology

The Service for the Mental Health of Children and Adolescents is an out-patient clinic that has been operating through the Community Center for Mental Health since 1982. Diagnostic assessment is the primary service provided. The therapeutic intervention that follows may include counselling or supportive intervention in the family, individual treatment for the child and psychosocial support. Special treatment for learning disabilities, speech disorders and delays in mental development are also provided. Interventions aiming at community awareness, as well as research projects, are part of the multidisciplinary team's work.

A 20-item multiple choice questionnaire, specifically developed for this study, was administrated during the first contact of the parents with the child psychiatry service of the Center. The questionnaire comprised of questions regarding the attitudes and beliefs of parents on children's psychotropic medication. Parents were asked whether they believe that psychotropic drugs are effective in the treatment of mental disorders of childhood, whether they have a therapeutic effect or only act by chance, whether or not they act through a modification of a biological abnormality in the brain, whether they cause dependence or harmful physical effects, whether they are overused etc. (the questionnaire is shown in Additional file 1).

In addition, the sex, age, educational level, place of residence, personal and family history of the parent as well as the presenting problem, sex, age and educational status of the child were recorded. With regard to parental educational level, we ranked it as low, medium or high corresponding to elementary, high school and university education, respectively.

The psychiatric diagnosis of the child was also noted. With respect to the diagnostic procedure, there is a standard practice in our service whereby each case is assessed independently and in cooperation by different members of the multidisciplinary team, according to the specific request and needs of each case. Finally, the case is presented in the weekly case conference, where all members of the multidisciplinary team are present (child psychiatrists, psychologists, social workers, speech, occupational and educational therapists). The final diagnosis, according to ICD-10 instructions [16] recorded in the patient's file, is a product of the team's consensus.

### Statistical analysis

Frequency tables were created and the chi-square test and Fisher's exact test were used for comparison of the participants' responses according to sex, educational level, age and gender of the child and use of medication.

## Results

A total of 134 out of 140 questionnaires were completed. Only two of the parents had former experience with a mentally disordered individual in the family. The demographic characteristics of the sample are presented in Table 1; the diagnosis concerning the child's presenting problem according to ICD-10 [13] is shown in Table 2, Table 3 shows the general views and beliefs of the respondents, and Table 4 shows the results concerning the opinion of the respondents about the efficacy of different modalities of treatment. Finally, Table 5 shows the respondents' opinion on the safety of psychotropic medication.

**Table 1:** Sociodemographic characteristics of the sample

		n = 134	%
***Parent's sex***	Men/women	22/112	16.6/83.3
***Parent's age (years)***	< 25	8	6.0
	25-45	106	79.0
	> 45	20	15.0
***Parent's educational level***			
	Low	41	30.6
	Middle	54	40.3
	High	39	29.0
***Child's sex***	Boy/girl	94/40	70.1/29.9
***Child's age (years)***	< 5	26	19.4
	6–12	80	59.7
	12–18	28	20.9

**Table 2:** Diagnosis according to ICD-10 classification system

	Age of child		
	≤ 5 Years n (%)	6–12 Years n (%)	12–18 Years n (%)

F32 depressive episode			4 (14.2)
F41 anxiety disorders		4 (5)	1 (3.5)
F43 reaction to severs stress and adjustment disorders		2 (2.5)	3 (10.7)
F60 specific personality disorders			2 (7.1)
F63 habit and impulse disorders		3 (3.7)	
F70 mild mental retardation	2 (7.6)	7 (8.7)	1 (3.5)
F71 moderate mental retardation		3 (3.7)	
F80 specific developmental disorders of speech and language	13 (50)	19 (23.7)	4 (14.2)
F81 specific developmental disorders of scholastic skills		23 (28.7)	10 (35.7)
F82 specific developmental disorders of motor function		3 (3.7)	
F83 mixed specific developmental disorders	3 (11.5)		
F84 pervasive developmental disorders	4 (15.3)	2 (2.5)	
F91 conduct disorders		2 (2.5)	1(3.5)
F92 mixed disorders of conduct and emotions	2 (7.7)	2 (2.5)	2 (7.1)
F93 emotional disorders with onset specific to childhood		10 (12.5)	8 (28.5)
F98 other behavioral and emotional disorders with onset usually occurring in childhood and adolescence	2 (7.7)		3 (10.7)

**Table 3:** General views and beliefs (n, %)

**Question**	**Yes n (%)**	**No n (%)**	**Don't know n (%)**
What's your general opinion on psychotropic medication?			
Do you believe that they cause sedation without curing?	62 (46.2)	21 (15.4)	51 (38.4)
Do they act therapeutically?	31 (23.1)	37 (27.7)	66 (49.2)
Do you believe that they have a common mechanism of action as tranquilizers?	33 (24.6)	60 (44.6)	41 (30.2)
Do you believe that they act on the brain correcting a biological abnormality responsible for the mental disease?	36 (27.2)	49 (36.4)	49 (36.4)
Do you believe that they are differentiated in categories (antipsychotics, antidepressants etc.) each with a different mechanism of action and efficacy?	112 (83.3)	6 (4.6)	16 (12.1)
What is your opinion about the use of psychotropic medication?			
Excessive use	50 (37.0)		
Normal use	19 (14.0)		
Low use	8 (6.0)		
I don't know	57 (43.0)		
Do you believe that psychiatrists unnecessarily use high doses of psychotropic medication?	27 (20.3)	34 (25.5)	73 (54.7)
Do you think that higher doses are more effective?	2 (1.6)	101 (75.0)	31 (23.4)
Do you take medication frequently (e.g. for headaches, insomnia etc)?	12 (9.1)	122 (90.9)	
Are you generally against medication?	95 (71.2)	39 (28.8)	
Do you fear psychotropic medication more than other medication?	107 (79.7)	27 (20.3)	

**Table 4:** Opinion on the efficacy of the treatment (n, %)

What is your opinion about the most effective treatment for the following disorders?	**In schizophrenia**	**In depression**	**In anxiety disorder**
Medication	9 (6.3)	8 (6.0)	4 (3.0)
Psychotherapy	68 (50.8)	88 (65.0)	92 (68.0)
Electroconvulsive therapy	2 (1.6)	0	0
Options 1 and 2	2 (1.6)	8 (6.0)	4 (3.0)
Don't know	53 (39.7)	30 (22.0)	34 (25.0)

**Table 5:** Opinion on the safety of psychotropic medication (n, %)

	**Yes**	**No**	**Don't know**
Do you believe that long-term use of psychotropic drugs could cause damage?	81 (60.3)	2 (1.3)	51 (38.1)
What do you fear most about prescribing psychotropic medication to children?			
They may cause damage to patient's health.	45 (33.3)		
They get used to them easier?	36 (26.7)		
They affect their learning abilities?	36 (26.7)		
If they start from early ages they will have greater problems in the future?	102 (75.5)		
Do you think that by taking psychotropic medication from early ages they would be more likely to develop drug addiction later?	86 (64.5)		

Additionally, the majority of parents (99; 74%) believe that psychotropic drugs are dangerous, but a clear differentiation exists among them. Only 18 (13%) believed that all psychotropic drugs are dangerous, while 81 (61%) limit the danger to just some categories. Two-thirds of the participants believe that psychotropic medication cause addiction (92 (69%) for antipsychotics, 99 (74%) for antidepressants, 76 (57%) for anxiolytics and 110 (82%) for hypnotics), while 23% did not express any opinion.

Statistical analyses reveal that significantly different responses were found between men and women on the question of whether they believe that there are different categories for psychotropic medication (Fisher's exact test, p = 0.042). The proportion of men (18.2%) who gave negative answers was greater than the same proportion of women (1.9%). No significant differences concerning parental opinion were found according to the gender of the child.

Possible differences in parental opinion according to the age of the child were also investigated. It was found that the proportion of parents who agree with the limitation to administrate psychotropics under special medical prescription increases as the age of the child increases. Specifically, 20% of the parents with children aged 2–7 years, 50% with children aged 7–12 years, and 75% with children aged older than 12 years responded that they agree with this limitation (Fisher's exact test, p = 0.008).

Additionally, a significantly lower proportion of the parents who were opposite to psychotropic medication take medication frequently themselves (4.3% vs. 21.1%). Furthermore, the responses to the question of whether psychotropic medication causes addiction differed between those who take medication frequently and those who do not (Fisher's exact test, p = 0.039). The proportion of positive responses was greater for those who take medication frequently (70% vs. 33.3%).

Parents' beliefs significantly differ according to educational level (χ^2 ^test, p = 0,037). The proportion of negative responses increases as the educational level increases (11.8% for low educational level, 45.8% for mid-level and 50.0% for high educational level).

## Discussion

The results of this study indicate that a significant proportion of parents have a negative opinion on psychotropic medication, and their beliefs differ from general findings regarding their safety.

Deeper socio-cultural beliefs seem to affect the acceptance (or non-acceptance) of medication. In the study of Schnittker [17], it was shown that African-Americans are more reluctant to take or to accept psychotropic medication for their children in comparison to the Caucasian population. In a recent study [18] it was shown that Caucasian race is associated with higher proportions of medication use among children in the Child Welfare System. African-American and Latino races were associated with lower proportions of medication use. Our sample was homogeneous regarding race and ethnicity (Greek) and the opinions on psychotropic medication are not statistically differentiated with parental age. The only significant difference found between men and women concerned the question of whether they believe that there are different categories of psychotropic medication. Women seemed to be better informed than men about this issue. Educational level only influenced the fear of drug addiction. Where the educational level was higher, parents seem to be closer to the scientific point of view and they did not fear the risk of addiction as much.

Our results show that most parents have a greater fear concerning their children taking psychotropic medication than for other types of medication. This is consistent with the study of Pappaport and Chubinsky [19], who found that while parents easily give cough medicine or antibiotics, they are afraid to give medication that might alter the behaviour or thinking of their children. Parental hesitation to administer psychotropic medication to their children may intervene with the treatment and affect treatment compliance. The high percentage of children that discontinue pharmacotherapy confirms that assertion [20]

According to our results parents seem to be aware of the distinction of psychoagents into four categories, but the majority of the respondents seem to not be well informed about the safety of psychotropic drugs. This contradiction is probably due to the fact that the structure of the question, concerning the distinction of psychoagents, led to the correct answer. Most of them are afraid of potential dependency, and are affected by anti-drug public opinion. The prevalence of the belief that antipsychotics (69%) and antidepressants (74%) cause addiction is considerable. The use of psychostimulants during childhood in order to treat ADHD (attention deficit hyperactivity disorder) had been accused of causing dependence and predisposition to the use of controlled substances. More recent research has shown that their use does not increase the possibility of substance abuse later in life [21,22]. In fact, another study has found that children with ADHD who received pharmacotherapy (methylphenidate) were less likely to use alcohol or substances later in their life, in comparison to children that presented with hyperactivity but did not receive pharmaceutical treatment [23].

Most parents consider psychotherapy as the most effective treatment for mental disorders, including childhood schizophrenia (65%). According to Pappaport and Chubinsky [19], parents accept pharmacotherapy only when behavioural and psychological interventions have been exhausted. They then experience a process of grief and the acceptance of pharmacotherapy seems to coincide with the acceptance of the psychiatric diagnosis. It is the final proof of what they fear. They realize that their child is suffering from a serious mental illness that might accompany them into adulthood.

A considerable proportion of respondents believe that there is overuse of psychotropic medication during childhood. In a study [24] involving 302 parents whose children were hyperactive, the erroneous opinions about the disorders and the methods of treatment became apparent. A total of 75% of the parents expressed the view that sugar and diet affected hyperactivity, 55% were reluctant for their children to use medication and 33% believed that there is an overuse of drugs in children with hyperactivity disorder.

Even though antipsychotics today occupy a significant place in child psychiatry [25], there is a recorded reluctance by the mothers to accept them as a treatment option during their child's first psychotic episode [26]. Reluctance in accepting pharmacotherapy is also presented by fathers of hyperactive boys [27]. Through identification mechanisms, they consider that treatment with methylphenidate separates their children from their peers and makes them different and isolated.

A high percentage of American parents (57%) accept pharmacotherapy when their child has expressed suicidal ideas and a smaller percentage in the case of disruptive behaviour (34,2%) or hyperactivity (29.5%). These differences are not dependent on socio-economic factors or educational level but on the trust in the doctor [14]. Hyperactivity and hallucinations/delusions are the main problems for teachers that could lead to the use of pharmaceutical treatment [28]. In our sample, there was no correlation between the parental attitudes and the severity of the child's problem.

In the United States, in a study involving 1387 subjects [29], it was found that psychotropic medication represents an effective treatment and less than half of the sample involved were concerned about safety. However, the majority did not want to use them. Several studies [30-32] point out that parents are not satisfied by the way in which information is given to them by doctors about the benefits and risks of pharmacotherapy. They wish to know all potential side effects of the medication, and do not appreciate the doctor's withholding information on the subject.

## Conclusion

Our results indicate that the opinions and beliefs prevalent in the generation that has now reached parenthood are not consistent with scientific knowledge. Their negative attitude indicates that there is a need for better mental heath education. Fear of psychiatric stigmatization and ignorance of the nature of mental disorders are also important factors in the establishment of this attitude, which threatens to rule out pharmacotherapy as a way of dealing with certain childhood mental disorders.

Child psychiatrists ought to scientifically inform the parents on the efficacy and safety of treatment, and then take into consideration the opinions and attitudes of the family. Only by paying attention to the desires, fears and beliefs of both parents and children will they be able to encourage and ensure compliance to treatment.

### Limitations

The findings of this study must be considered under the following limitations: First, it is a descriptive study based on a relatively small sample where a new measure has been used. Concerning the structure of the questionnaire, it is possible that for some items the wording may be leading to certain answers. Also, because of the design of the study, the possible correlation of psychopathological severity of the child and parental attitude towards psychotropic medication was not investigated. Finally, it must be noted that the proportion of men who participated in the study was much lower than that of women.

## Competing interests

The author(s) declare that they have no competing interests.

## Authors' contributions

The authors all contributed equally to the manuscript, and both were involved in the drafting of the manuscript and have given the final approval of the submitted version.

## Supplementary Material

Additional file 1 questionnaire used in Word format. Click here for file
