# Supplementary material for: Parental attitudes and opinions on the use of psychotropic medication in mental disorders of childhood
Source: Ann Gen Psychiatry. 2007 Nov 15;6:32. doi: 10.1186/1744-859X-6-32 (PMC2206023; doi:10.1186/1744-859X-6-32)
Supplement: Additional file 1 — questionnaire used in Word format. [file 1744-859X-6-32-S1.doc]

**The questionnaire**

Psychiatric Department, University of Athens

Eginitio Hospital

Laboratory of Clinical Psychopharmacology

Parent completing the questionnaire

1. Sex: Male Female 5

2. Age: <25 25 - 45 >45 6

3. Parent’s Educational Level (years of studies)……………………………………..…………………………… 8

4. Place of residence ………………………………………………….. 9

5. Have you ever had a member of your family suffering from mental disorder?

Yes No 10

Child’s Record

6. Sex: Boy Girl 11

7. Age: 12

8. Years of studies:……………………………………………………… 13

9. Place of Residence:………………………………………………… 14

Please answer the following questions regarding your opinion on psychotropic medication.

Please answer all the questions.

**1.** What’s your general opinion on psychotropic medication?

a. Do you believe that they cause sedation without curing?

YES NO I don’t know 15

b. Do they act therapeutically? YES NO 16

**2.** Do you believe that they have a common mechanism of action as tranquilizers? YES NO I don’t know 17

**3.** Do you believe that psychotropics are differentiated in categories?

(e.g. antidepressants, anxiolytics, hypnotics and anti-psychotics) each one with different mechanism of action and efficacy?

YES NO I don’t know 18

**4.** Do you believe that psychotropics act on the brain correcting a biological abnormality responsible for the medical disease?

YES NO I don’t know 19

**5.** Do you believe that the psychotropic drugs cause addiction?

YES NO I don’t know

If YES, do they all or some of them cause addiction, which of them?

(underline)

1. All of them
2. Antipsychotics
3. Antidepressants
4. Anxiolytics
5. Hypnotics 21

**6.** Do you believe that by taking psychotropic medication the user will become addicted and therefore be unable to stop taking it?

YES NO I don’t know 22

**7.** What is your opinion about the use of psychotropic medication?

(underline)

1. Excessive use
2. Normal use
3. Low use
4. I don’t know

23

**8.** Do you believe that long-term use of psychotropic drugs could cause damage (e.g. on the brain, kidneys, liver etc)

YES NO I don’t know 24

If YES, all or some of them? All Some of them 25

**9.** Do you believe that psychotropic drugs are dangerous?

1. Yes, all of them

2. Yes, some of them

3. No

4. I don’t know

26

**10.** Do you think that psychiatrists use unnecessarily high doses of psychotropic medications?

YES NO I don’t know 27

**11.** Do you think that higher doses are more effective?

YES NO I don’t know 28

**12.** Do you think that in some cases long-term use of psychotropic medication is necessary, so that the patient does not relapse?

YES NO I don’t know 29

**13.** Would you prefer drug treatment instead of the more effective electroconvulsive therapy?

30

Yes No I don’t know

**14.** Which is your opinion about the most effective treatment for the following disorders?

(underline)

1. In Psychosis 1. Medication

2. Psychotherapy

3. Electroconvulsive therapy

4. I don’t know 31

2. In Depression 1. Medication

2. Psychotherapy

3. Electroconvulsive therapy

4. I don’t know 32

3. In Anxiety 1. Medication

2. Psychotherapy

3. Electroconvulsive therapy

4. I don’t know 33

**15.** Do you take medication frequently? (e.g. for headaches, insomnia etc)

YES NO 34

**16.** Are you generally against medication? Yes No 35

**17.** Do you fear psychotropic medication, more that other medication?

YES NO 36

**18.** What do you fear most about prescribing psychotropic medication to children?

a. They may cause damage to patient’s health 37

b. They get used to them easily 38

c. They affect their learning abilities 39

d. If they start at early ages they will have greater problems

in the future 40

**19.** Do you believe that the psychotropic drugs may cause more damage to children, due to their age?

YES NO I don’t know 40

**20.** Do you think that by taking psychotropic medication from early ages they would be more likely to develop drug addiction later?

YES NO I don’t know 41
